# *In vivo* determination of protective antibody thresholds for SARS-CoV-2 variants using mouse models

**DOI:** 10.1080/22221751.2025.2459140

**Published:** 2025-01-24

**Authors:** Peilan Wei, Ruoxi Cai, Lu Zhang, Jingjun Zhang, Zhaoyong Zhang, Airu Zhu, Hai Li, Zhen Zhuang, Lan Chen, Jiantao Chen, Yuting Zhang, Xinyi Xiong, Bin Qu, Jianfen Zhuo, Tian Tang, Yuanyuan Zhang, Lei Chen, Qier Zhong, Zhiwei Lin, Xindan Xing, Fang Li, Qingtao Hu, Jun Dai, Yongxia Shi, Jingxian Zhao, Jincun Zhao, Yanqun Wang

**Affiliations:** a State Key Laboratory of Respiratory Disease, National Clinical Research Center for Respiratory Disease, Guangzhou Institute of Respiratory Health, the First Affiliated Hospital of Guangzhou Medical University, Guangzhou, People’s Republic of China; b Guangzhou National Laboratory, Guangzhou, People’s Republic of China; c Health and Quarantine Laboratory, State Key Laboratory of Respiratory Disease of Guangzhou Customs District Technology Center, Guangzhou, People’s Republic of China; d NHC Key Laboratory of Medical Virology and Viral Diseases, National Institute for Viral Disease Control and Prevention, Chinese Center for Disease Control and Prevention, Beijing, People’s Republic of China; e Shanghai Institute for Advanced Immunochemical Studies, School of Life Science and Technology, ShanghaiTech University, Shanghai, People’s Republic of China; f GMU-GIBH Joint School of Life Sciences, Guangzhou Medical University, Guangzhou, People’s Republic of China; g Institute for Hepatology, National Clinical Research Center for Infectious Disease, Shenzhen Third People’s Hospital, Shenzhen, People’s Republic of China; h The Second Affiliated Hospital, School of Medicine, Southern University of Science and Technology, Shenzhen, People’s Republic of China

**Keywords:** SARS-CoV-2, protective antibody, threshold, neutralizing titre, mouse model, *in vivo*

## Abstract

Neutralizing antibody titres have been shown to correlate with immune protection against COVID-19 and can be used to estimate vaccine effectiveness. Numerous studies have explored the relationship between neutralizing antibodies and protection. However, there remains a lack of quantitative data directly assessing the minimum effective protective neutralizing antibody titre in *in vivo*. In this study, we utilized eight cohorts of participants with diverse immune backgrounds for evaluation of protective antibody response. To precisely assess the lower threshold of neutralizing antibody titres required for effective protection against SARS-CoV-2 infections, we employed plasma adoptive transfer from different cohorts into mice. This study demonstrated that neutralizing titres in the plasma of recipient mice correlated well with those in human donors, and a positive linear correlation was observed between the human and mouse recipients of transferred plasma neutralizing titre. A pseudotyped virus neutralizing titres greater than 7 was identified as the minimum threshold necessary to reduce viral titres in infected mice, establishing a crucial baseline for effective protection. Furthermore, despite the variability in immune backgrounds, these diverse cohorts’ plasma exhibited a similar neutralizing antibody threshold necessary for protection. This finding has significant implications for vaccine design and the assessment of immune competence.

## Introduction

SARS-CoV-2 vaccines are primarily designed to stimulate the production of neutralizing antibodies against specific regions of the spike protein, particularly the receptor-binding domain (RBD). The continual antigenic drift within the spike protein of variants necessitates regular updating of SARS-CoV-2 vaccine candidates [1]. Understanding the protective thresholds for these vaccines is crucial for vaccine evaluation and development, especially given the gradually decline in antibody levels over time following natural infection and vaccination, as well as the emergence of SARS-CoV-2 variants. The protective threshold value of a vaccine refers to the level of immune response, including neutralizing antibody titres, binding antibody levels, or T cell response, that correlates with protection against infection. Due to the ability to block virus attachment and entry into host cells, neutralizing antibodies are recognized as the most potent mediators of resistance to SARS-CoV-2 infection, and are considered the primary immune correlate of protection. Multiple reports indicate that neutralizing antibody titre has become the most critical parameter for evaluating vaccine efficacy and determining protective thresholds [2].

Neutralizing antibodies target the virus, preventing it from infecting cells. The haemagglutination inhibition (HI) assay is the standard method to assess immunity against influenza virus, measuring the ability of antibodies to block the virus from binding to receptors on red blood cells and preventing agglutination [3]. Previous studies have shown that an HI titre of 1:40 is generally considered to correlate with approximately 50% protection against influenza infection in healthy adults [4], and higher titres of neutralizing antibodies correlate with greater protection. Although the exact threshold can vary depend on different variants. Similar results have been observed with SARS-CoV-2 [5]. Recent studies suggest that a neutralizing antibody titre of 1:80 or higher is considered indicative of a protective immune response against SARS-CoV-2 [6], though this can vary depending on the assay and population studied. Another study suggested that a neutralizing antibody titre of 1:250 or higher was associated with 80% protection against symptomatic COVID-19 [2]. However, lower titres may also provide partial protection [7], the minimum effective protective neutralizing antibody titre for SARS-CoV-2 variants *in vivo* is still unclear.

These protective threshold values are often determined through clinical trials and epidemiological studies, which require long-term follow-up and are challenging. However, using adoptive transfer in animal models can rapidly determine protective thresholds, providing valuable reference data for human neutralizing antibody protective threshold studies [8,9]. Mouse models, especially those genetically modified to express human ACE2 (hACE2), the receptor for SARS-CoV-2, have been instrumental in studying the virus and evaluating vaccine efficacy. Previous studies have shown that neutralizing antibody titres of 1:40–1:160 have been associated with protection against SARS-CoV-2 in mice [10,11]. However, a major unresolved question is still the lack of an accurate protective antibody threshold and the minimum effective protective neutralizing antibody titre threshold for SARS-CoV-2 variants in mouse model. In addition, mice with high levels of antibody-dependent cellular cytotoxicity (ADCC) and antibody-dependent cellular phagocytosis (ADCP) mediating antibodies also showed enhanced clearance of SARS-CoV-2, demonstrating the importance of ADCP and ADCC in protective immunity [12,13]. In this study, we identify the neutralizing antibody titre threshold of approximately pVNT_50_ = 7 as the minimum required for effective protection against SARS-CoV-2 *in vivo* using mouse models. The finding provides critical insights into the immune mechanisms that confer protection against SARS-CoV-2 and has significant implications for vaccine design and public health strategies, particularly in the context of emerging variants.

## Materials and methods

### Cells and viruses

Vero E6 cell lines (CRL-1586) used in this study were acquired from ATCC. Recombinant HEK293T cells stably overexpressing the SARS-CoV-2 spike protein were purchased from GenScript (M00804). The 293T-hACE2 cell line was generated by transducing HEK293T cells with human angiotensin-converting enzyme 2 (hACE2) and subsequently selecting for stable cell lines. SARS-CoV-2 variant XBB.1.5 was isolated from COVID-19 patients in Guangdong, China. All experiments involving authentic SARS-CoV-2 were conducted in the Biosafety Level 3 (BSL-3) Laboratory at the Guangzhou Customs District Technology Center.

### Samples enrolled in this study

Eight groups of participants were enrolled in this study. In detail, (1) Healthy donor group (HD group): This group comprised 15 plasma samples collected from healthy individuals before 2019. (2) Convalescent group (WT group): This group included 15 plasma samples from convalescent patients infected with wild-type SARS-CoV-2 during the first wave of the pandemic. (3) BA.5 breakthrough infection group (BA.5 group): This group consisted of 15 plasma samples obtained from individuals who had received three doses of inactivated SARS-CoV-2 vaccine CoronaVac prior to BA.5 breakthrough infections. (4) XBB.1 breakthrough infection group (XBB.1 group): This group consisted of 15 plasma samples from patients who had received three doses of inactivated SARS-CoV-2 vaccine followed by BA.5 breakthrough infection and XBB.1 reinfection. (5) mRNA vaccine homologous booster group (B-B-B group): This group included 15 donors who received three doses of mRNA vaccine BNT162b2. (6) Inactivated/aerosolized Ad5-nCoV heterologous booster group (I-I-A group): This group included 15 donors who received two doses of inactivated SARS-CoV-2 vaccine followed by aerosolized Ad5-nCoV vaccine Convidecia. (7) Inactivated/mRNA vaccine heterologous booster group (I-I-B group): This group comprised 15 donors who received two doses of inactivated SARS-CoV-2 vaccine followed by one dose of mRNA vaccine. (8) Inactivated/aerosolized Ad5-nCoV heterologous second booster group (I-I-I-A group): This group consisted of 15 donors who received three doses of inactivated SARS-CoV-2 vaccine followed by aerosolized Ad5-nCoV vaccine.

### Production and titration of SARS-CoV-2 pseudovirus

SARS-CoV-2 pseudotyped viruses were prepared using the vesicular stomatitis virus (VSV) pseudotyping system [14]. Plasmids encoding the WT and BA.5 spike protein were transfected into 293T cells, which were subsequently infected with rVSV-ΔG-luc after 24 h. Viral supernatants were collected 24 h post-infection and stored at −80°C. XBB.1, XBB.1.5, XBB.1.16, XBB.2.3 and EG.5 were purchased from DaRui Bioscience. The 50% tissue culture infectious dose (TCID_50_) was determined using the Reed–Muench method. For titration, an initial 10-fold dilution of the pseudovirus was performed in 96-well plates, followed by serial 5-fold dilutions. Trypsin-treated 293T-hACE2 cells were then added. After 24 h of incubation, 50 μL luciferase substrate was added, and luminescence was measured using a microplate luminometer. Wells with luminescence values ten times higher than the background were considered positive.

### Pseudovirus neutralization assay

A pseudovirus neutralization assay was performed using Vesicular Stomatitis Virus (VSV) pseudotyped with SARS-CoV S protein as previously reported [14,15]. 50 μL of SARS-CoV-2 pseudovirus (3 × 10^4^ TCID_50_/mL) were incubated with serially diluted plasma (3-fold) at 37°C for 1 h. Subsequently, 100 μL of 293T-hACE2 cell suspension was added to the mixtures. After 24 h of incubation at 37°C, the supernatant was discarded, and 50 μL Bright-Glo substrate was added to each well for the detection of luminescence using a microplate luminometer (Bioteck).

### Focus formation assay (FFA)

FFA assay was used for the detection of lung viral titre as previously reported with some modification [16]. Vero E6 cells were seeded into 96-well plates and incubated overnight. Lung homogenates were serially diluted and added to the cells, followed by 24 h incubation at 37°C with 5% CO_2_. The plates were then fixed with 4% paraformaldehyde for 30 min. Vero E6 cells were permeabilized with 0.2% Triton X-100 and blocked with 1% BSA in PBS for 30 min. At last, the cells were stained with rabbit anti-SARS-CoV-2 N protein polyclonal antibody at 37°C for 1 h. After washing the plates three times with PBST (PBS, 0.05% Tween 20), an HRP-labelled goat anti-rabbit secondary antibody (Cat. No. 109-035-088, Jackson) was added. Foci were visualized using TrueBlue Peroxidase Substrate (KPL) and counted using an ELISPOT reader (Cellular Technology Ltd).

### Antibody-dependent cellular cytotoxicity (ADCC) and antibody-dependent cellular phagocytosis (ADCP) assay

HEK293T cells expressing SARS-CoV-2 wild-type spike protein (GenScript) were used as target cells, and Jurkat-FcγRIIIa-V158 Effector Cells and Jurkat-FcγRIIA-H131 cells (Vazyme) were respectively used as Antibody-dependent cellular cytotoxicity (ADCC) and Antibody-dependent cellular phagocytosis (ADCP) effector cells as previously reported [17]. Target cells, effector cells, and gradient diluted plasma were mixed in white 96-well plates and incubated for 6 h in cell culture incubator. Following incubation, Bright-Lite Luciferase Assay Solution was added and incubated at room temperature for 1 min. Luminescence was measured using a Tecan Microplate Reader. Experiments were performed in duplicate, and results are presented as mean ± SEM.

### Plasma adoptive transfer in mouse

Each C57BL/6 mouse (female, aged 5–7 weeks) received a single intravenous injection of 200 µL plasma, then blood samples were collected at different time points post-injection via retro-orbital venous plexus, including 0.5 h, 6 h, 12 h, 24 h, 2 d, 4 d, 7 d, 10 d, 13 d, 16 d, 19 d, 22 d, 25 d, and 28 d. Enzyme-linked immunosorbent assay (ELISA) was used to analyze SARS-CoV-2-specific IgG antibodies in mouse sera against the SARS-CoV-2 WT strain spike (S). ELISA was performed on the sera samples as previous report [12]. Plates were read using a BioTek Epoch microplate reader at an optical density (OD) of 450 nm. This method was performed to assess the half-life of human plasma and examine the correlations between anti-SARS-CoV-2 IgG levels at different time points post-injection.

### Challenge of immune plasma-transferred mice with SARS-CoV-2 variant

K18-hACE2 transgenic mice, aged 5–7 weeks, were injected intravenously with 200 µL of plasma per mouse 6 h before being challenged with 5 × 10^4^ FFU of SARS-CoV-2 Omicron XBB.1.5. The control group received plasma from healthy donors followed by the same SARS-CoV-2 challenge dose. Lung tissues were harvested for histopathological analysis and lung viral titres were assessed on day 2 and 3 post-challenge using focus forming assay (FFA). In addition, lungs were fixed in zinc formalin, embedded in paraffin, tissue sections (∼4 µm each) were stained with haematoxylin and eosin for examination by light microscopy. All experiments with authentic SARS-CoV-2 were performed at the Guangzhou Customs District Technology Center BSL-3 Laboratory.

### Ethics approval

The procedures for plasma adoptive transfer and challenge assays in mice were approved by the Institutional Animal Care and Use Committees at the First Affiliated Hospital of Guangzhou Medical University (2023-599). This study was authorized by the Ethics Committee at the First Affiliated Hospital of Guangzhou Medical University (2022030), all participants provided written informed consent.

### Statistics and reproducibility

Statistical analyses were performed using GraphPad Prism 8 software. Detailed information about the statistical tests utilized can be found in the figure legends. Non-linear regression analysis was employed to calculate the IC_50_ values for pseudovirus neutralization titres. The Mann–Whitney test was used to evaluate the significance of differences, with a *p*-value of <0.05 considered to be statistically significant (* *p* < 0.05, ** *p* < 0.01, *** *p* < 0.001, and ****p < 0.0001).

## Results

### Study populations

**Figure 1.**
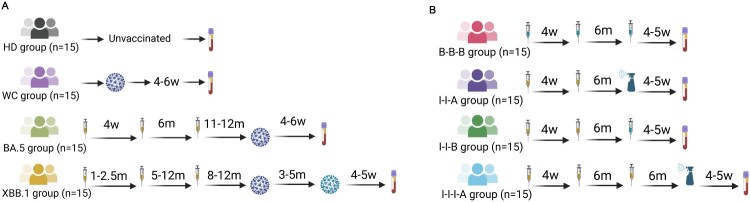
Cohort details and timelines for plasma sample collection. (a) The cohorts and timing of plasma sample collection from different groups in the study: healthy donors (HD), convalescents infected with the wild-type strain (WT), and convalescents from BA.5 and XBB.1 breakthrough infections (BA.5, XBB.1). (b) The cohorts and timing of plasma sample collection from vaccine recipients who received either homologous (B-B-B) or heterologous (I-I-A, I-I-B, I-I-I-A) booster vaccinations. Here, “I” denotes the inactivated vaccine CoronaVac, “B” denotes the mRNA vaccine BNT162b2, and “A” denotes the aerosolized vaccine Convidecia (Ad5-nCoV). Time points are indicated by “w” for week and “m” for month. Created in https://BioRender.com.

As previously reported that neutralizing antibody titre correlates with immune protection and is highly predictive of immune protection [5,18,19]. The changing landscape of COVID-19 vaccination highlights the need for accurate evaluation of protective antibody immune response against SARS-CoV-2 variants. Herein, eight cohorts of participants with diverse immune backgrounds were enrolled for the evaluation of plasma neutralizing activity in this study (Figure 1), including healthy donor group (HD), convalescent group infected with wild-type SARS-CoV-2 strain (WT), BA.5 breakthrough infection group (BA.5), XBB.1 breakthrough infection group (XBB.1), mRNA vaccine homologous booster group (B-B-B), inactivated/mRNA vaccine heterologous booster group (I-I-B), inactivated/aerosolized Ad5-nCoV heterologous booster group (I-I-A) and inactivated/aerosolized Ad5-nCoV heterologous second booster group (I-I-I-A). All plasma was collected around 4–5 weeks after breakthrough infection or booster vaccination.

### Evaluation of protective antibody response against SARS-CoV-2 variants among populations with diverse immune backgrounds

**Figure 2.**
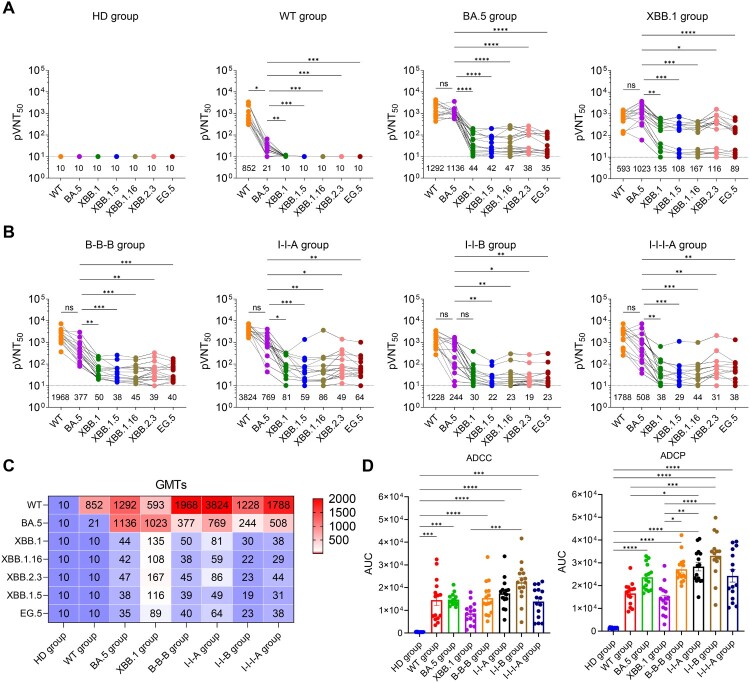
Evaluation of protective antibody response against SARS-CoV-2 variants among populations with diverse immune backgrounds. (a) Plasma neutralization titres against Omicron variants in healthy donors (HD), wild-type strain convalescents (WT), BA.5 and XBB.1 breakthrough infection convalescents (BA.5, XBB). Geometric mean neutralizing titres (GMTs) are displayed at the bottom of the plots. (b) Neutralizing titres against various SARS-CoV-2 pseudovirus in plasma from vaccines who received homologous (B-B-B) or heterologous (I-I-B, I-I-A, I-I-I-A) booster vaccination. (c) Heatmap showing the GMTs against prototype and diverse Omicron subvariants from healthy donors, vaccines, convalescents and breakthrough infection patients. (d) Antibody-dependent cellular cytotoxicity (ADCC) and antibody-dependent cellular phagocytosis (ADCP) activity induced in different immune groups. AUC stands for area under curve for the ADCC and ADCP responses, and was calculated using Prism 8 (GraphPad). Kruskal–Wallis test with Dunn’s multiple comparisons test was used for evaluating differences among the experimental groups. Error bars represent mean ± SEM, *p*-values are displayed as ns for *p* > 0.05, *p < 0.05, **p < 0.01, ***p < 0.001, and ****p < 0.0001.

We first evaluated the neutralizing activity of plasma from vaccinated individuals and convalescents against several representative SARS-CoV-2 variants, including WT, BA.5, XBB.1, XBB.1.5, XBB.1.16, XBB.2.3, and EG.5. As shown in Figure 2(a), neutralization titres against XBB.1, XBB.1.5, XBB.1.16, XBB.2.3, and EG.5 were below the limit of detection (pVNT_50_ value of 10) in both HD and WT groups. XBB and subvariants exhibits significant immune evasion potential, the neutralizing titres against variant EG.5 were markedly reduced as compared to the BA.5. The vaccine groups, including B-B-B, I-I-A, I-I-B, I-I-I-A, showed a similar profile to that observed in breakthrough infection group (Figure 2b). Multiple booster vaccination strategies failed to elicit high neutralizing antibody titre against the newly emerged Omicron subvariants with pVNT_50_ GMTs (geometric mean titres) < 100. Similar trends were observed for both vaccine- and infection-induced plasma, regardless of the vaccination status (Figure 2c and Figure S1). These results highlight the importance of updating vaccine strains to match circulating variants to ensure effective protection in the future.

Antibody-dependent cellular cytotoxicity (ADCC) and antibody dependent cellular phagocytosis (ADCP) functions induced by plasma antibodies are integral to the protective immune response against SARS-CoV-2 infection. They contribute significantly to viral clearance, reduction of disease severity, and overall immune protection. In this study, ADCC and ADCP activity against SARS-CoV-2 wild-type strain were characterized using reporter bioassay with Jurkat-FcγRIIIa-V158 and Jurkat-FcγRIIa-H131 cells as effector cells, respectively, and luciferase substrate as reacting regent for monitoring Fc receptor signalling (Figure 2d and Figure S2). Both plasma from vaccinated individuals and convalescent patients showed strong ADCC activity. Similar results were observed in the assessment of ADCP activity. Different COVID-19 vaccine immunization strategies elicit varying levels of ADCC and ADCP responses, which can impact the effectiveness of the immune response against SARS-CoV-2, thereby influencing viral clearance and disease outcomes [20].

### Establishment of plasma adoptive transfer for protective antibody evaluation *in vivo*

**Figure 3.**
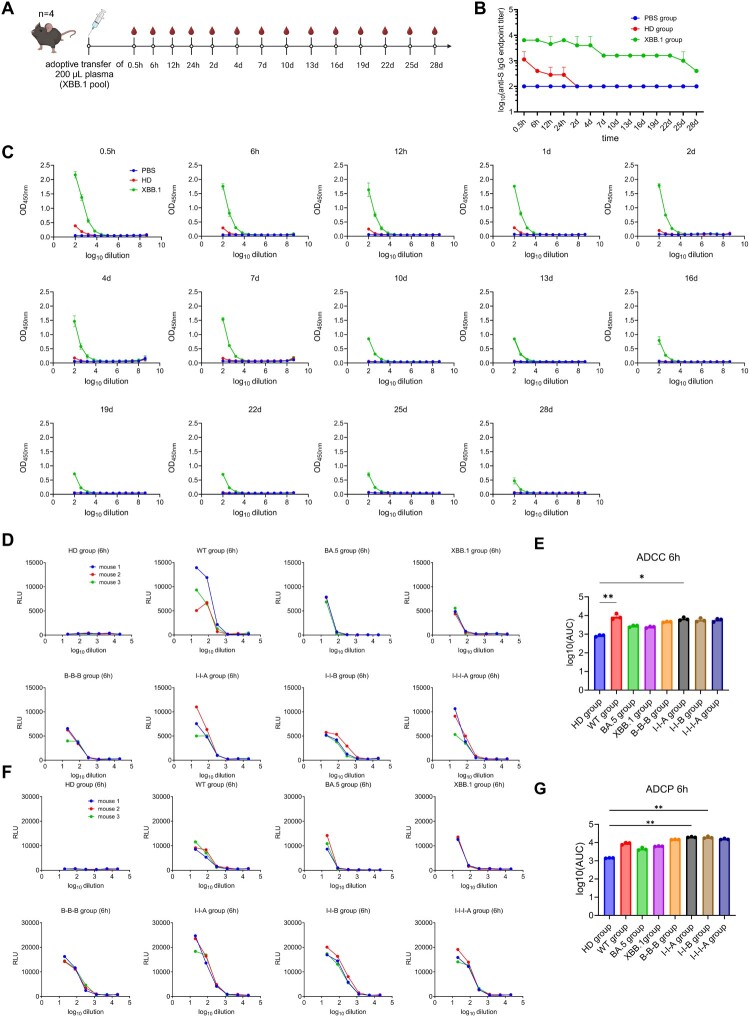
Dynamic monitoring of SARS-CoV-2 specific antibody titres in mice recipients of transferred plasma. To determine the pharmacokinetics (PK) of human plasma in mouse, mice were administered 200 µL of plasma each via intravenous injection. For the control groups, 200 µL of phosphate-buffered saline or plasma from healthy donors was injected. Blood samples were collected at different time points post-injection and used for spike-specific human antibody and Fc-dependent antibody effector functions (ADCC and ADCP) analysis. (a) Adoptive transfer strategy. Created in https://BioRender.com. (b, c) Dynamic monitoring of antibody titres in mice at 0.5 h, 6 h, 12 h, 24 h, 2 d, 4 d, 7 d, 10 d, 13 d, 16 d, 19 d, 22 d, 25 d, and 28 d after plasma adoptive transfer. Diluted mice sera were used for SARS-CoV-2 spike-specific IgG antibodies using ELISA assay. (d, e) Antibody-dependent cellular cytotoxicity effect (ADCC) analysis in mice 6 h post adoptive. (f, g) Antibody-dependent cellular phagocytosis effect (ADCP) analysis in mice 6 h post adoptive transfer. AUC stands for area under curve for the ADCC and ADCP responses, and was calculated using Prism 8 (GraphPad). Kruskal–Wallis test with Dunn’s multiple comparisons test was used for evaluating differences among the experimental groups. Error bars represent mean ± SEM, *p-*values are displayed as ns for *p* > 0.05, *p < 0.05, **p < 0.01 ***p < 0.001, and ****p < 0.0001.

Rapid development of predictive models of immune protection against COVID-19 is crucial to identify correlates of protection. To assess the lower threshold of neutralizing antibody titres required to confer effective protection against SARS-CoV-2 infections, we employed a plasma adoptive transfer method *in vivo* using mouse models. In detail we transferred immune plasma from XBB.1 breakthrough infection group to naïve, non-immunized C57BL/6 mice as illustrated (Figure 3a). Mice were administered 200 µL of plasma each via intravenous injection. For the control groups, 200 µL of plasma from healthy donors (HD) was injected. Blood samples were collected at various time points post-injection for analysis of spike-specific human antibody and Fc-dependent antibody effector functions (ADCC and ADCP). Results indicated that the half-life of spike-specific human antibodies in mice is approximately 4–7 days, as determined by the kinetics of spike-specific antibody endpoint titres (Figure 3b,c), consistent with a previous study [21], which facilitates the assessment of antibody persistence and effectiveness.

Additionally, we evaluated ADCC and ADCP activity against SARS-CoV-2 in mouse sera collected 6 h after transfer. All eight groups participated in this assessment, with each group transferring three plasma samples to separate mice. Results indicated that, following adoptive transfer of immune plasma, mice exhibited significant ADCC (Figure 3d,e) and ADCP (Figure 3f,g) activity against SARS-CoV-2 compared to the HD group. Although previous studies have shown that human IgG can bind to murine Fc receptors, with generally lower affinity compared to human Fc receptors [21], the murine Fc receptor still facilitates some degree of protection for human IgG antibodies.

### Relationship between pVNT_50_ in human plasma and in mouse recipients of transferred plasma

**Figure 4.**
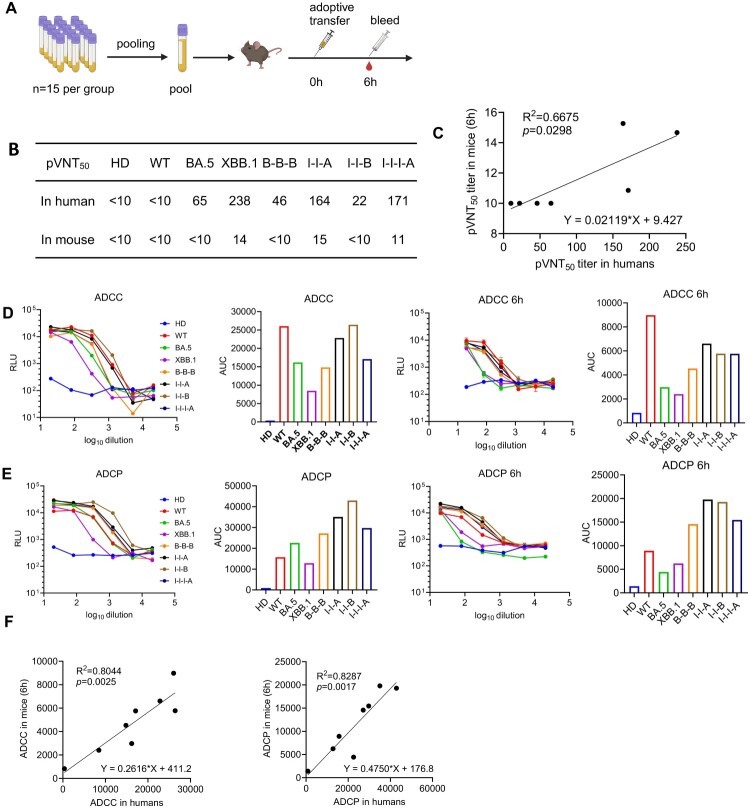
Relationship between pVNT_50_ in human plasma and in mouse recipients of transferred plasma. (a) Plasma from each group was pooled into one sample for subsequent protective evaluation, here is the adoptive transfer of immune plasma pools. Created in https://BioRender.com. (b) pVNT_50_ titre against XBB.1.5 pseudovirus in human plasma pools and in mouse recipients of transferred plasma. (c) Relationship between pVNT_50_ in human plasma pools and in mouse recipients of transferred plasma. Spearman correlation analysis was performed to analyze the correlation between pVNT_50_ titre in humans and mouse plasma. Antibody-dependent cellular cytotoxicity effect (ADCC) (d) and antibody-dependent cellular phagocytosis effect (ADCP) (e) activity in human plasma pools and mouse recipients of transferred plasma (6 h post injection). RLU values were expressed as mean ± SEM. (f) Relationship between ADCC, ADCP in human plasma pools and in mouse recipients of transferred plasma. Spearman correlation analysis was performed to analyze the correlation.

To ensure representative sampling, plasma from each group was pooled into one sample for subsequent protective evaluation in this study (Figure 4a). To determine the relationship between pVNT_50_ in human plasma and in mouse recipients of transferred plasma, we transferred 200 μL of plasma from each pooled sample to C57BL/6 mice. Considering the plasma half-life and prior research [22], blood sample was collected at 6 h post-injection for analysis of neutralizing titre (Figure 4b) and Fc-dependent antibody effector functions, including ADCC (Figure 4d) and ADCP (Figure 4e). SARS-CoV-2 pseudotype neutralization assay was performed against the representative stain XBB.1.5. Results indicated that neutralizing titres in the plasma of recipient mice correlated well with titres in human plasma, and a positive linear correlation was observed between the human and mouse recipients of transferred plasma neutralizing titre (Figure 4c). Similar trends were observed in ADCC and ADCP activity following plasma adoptive transfer (Figure 4f). Given that transferring 200 μL of plasma to a 20-gm mouse is equivalent to transferring 600 mL plasma to a 60 kg patient (calculated on a per kg basis), these data provide a framework for protective efficacy evaluation *in vivo*.

### Determination of protective antibody thresholds for SARS-CoV-2 *in vivo*

**Figure 5.**
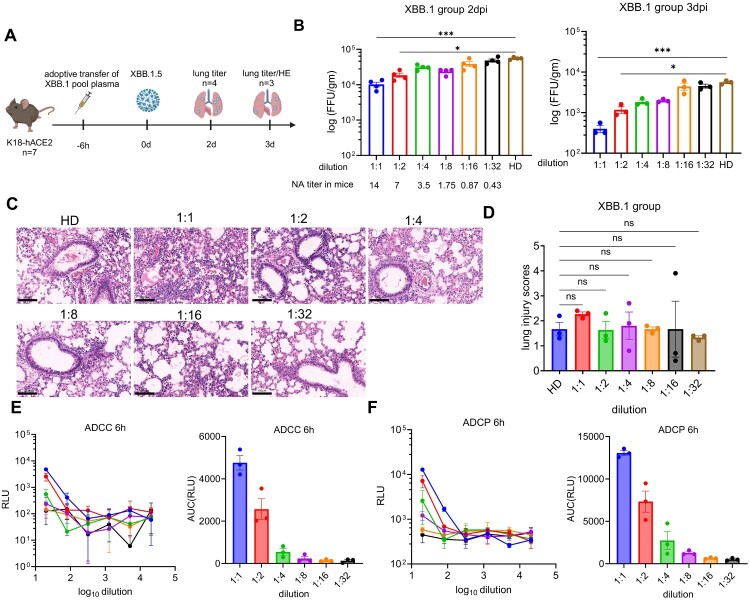
Determination of the minimum antibody level required to confer effective protection against SARS-CoV-2 infection *in vivo*. (a) Timeline for plasma adoptive transfer and live SARS-CoV-2 challenge experiment. Created in https://BioRender.com. We transferred 200 μL of 2-fold serial dilution of plasma from XBB.1 breakthrough infection group pool to K18-hACE2 mice sensitized for SARS-CoV-2 infection. Six hours after plasma transfer, challenge the mice with SARS-CoV-2 XBB.1.5. (b) Lung viral titres were measured at 2 and 3 dpi. Plasma dilutions and corresponding neutralizing titres in mice sera were labelled. (c) Representative Haematoxylin-eosin (HE) staining of lungs from C57BL/6 mice harvested at 3 dpi. Scale bar = 100 μm. (d) Summary of histology score (necrotic cellular debris, mononuclear, oedema, and neutrophils) at 3 dpi. (e) ADCC and ADCP responses in mouse recipients of transferred plasma. AUC stands for area under curve for the ADCC and ADCP responses. Kruskal–Wallis test with Dunn’s multiple comparisons test was used for evaluating differences among the experimental groups. Error bars represent mean ± SEM, *p*-values are displayed as ns for *p* > 0.05, *p < 0.05, **p < 0.01, ***p < 0.001, and ****p < 0.0001*.*

To further assess the minimum antibody level required for effective protection against SARS-CoV-2 infection, we transferred 200 μL of 2-fold serial dilution of plasma from XBB.1 breakthrough infection group pool to K18-hACE2 mice sensitized for SARS-CoV-2 infection. Six hours after plasma transfer, challenge the mice with SARS-CoV-2 XBB.1.5 (5 × 10^4^ FFU/mouse) (Figure 5a). Lungs were homogenized at day 2 and 3 p.i. to obtain virus titres (Figure 5b). The results suggest that a pVNT_50_ > 7 (plasma dilution ratio = 1:2) was required to reduce virus titres in infected mice and determined as the lower threshold of neutralizing antibody titres required to confer effective protection. Mouse pVNT_50_ titres in the sera correlated inversely with virus titres in the mice lungs, and higher titres of neutralizing antibodies correlate with greater protection.

Haematoxylin and eosin-stained lung sections from SARS-CoV-2-infected K18-hACE2 mice revealed extensive lung pathology, marked by significant inflammatory cell infiltration around blood vessels and bronchi. However, no notable reduction in lung injury was observed in the mice that received adoptive plasma transfer compared to the control group (Figure 5c,d), indicating that the minimum antibody levels pVNT_50_ of >7 (dilution 1:2) insufficient to reduce pulmonary pathological damage, indicating that higher neutralizing antibody titres are needed for effective prevention of lung damage. Additionally, ADCC and ADCP responses in paired human and adoptive transfer mice sera were strongly correlated, with the minimum antibody levels corresponding to approximately 2000 and 5000 relative light units (RLU) for ADCC and ADCP, respectively (Figure 5e,f).

**Figure 6.**
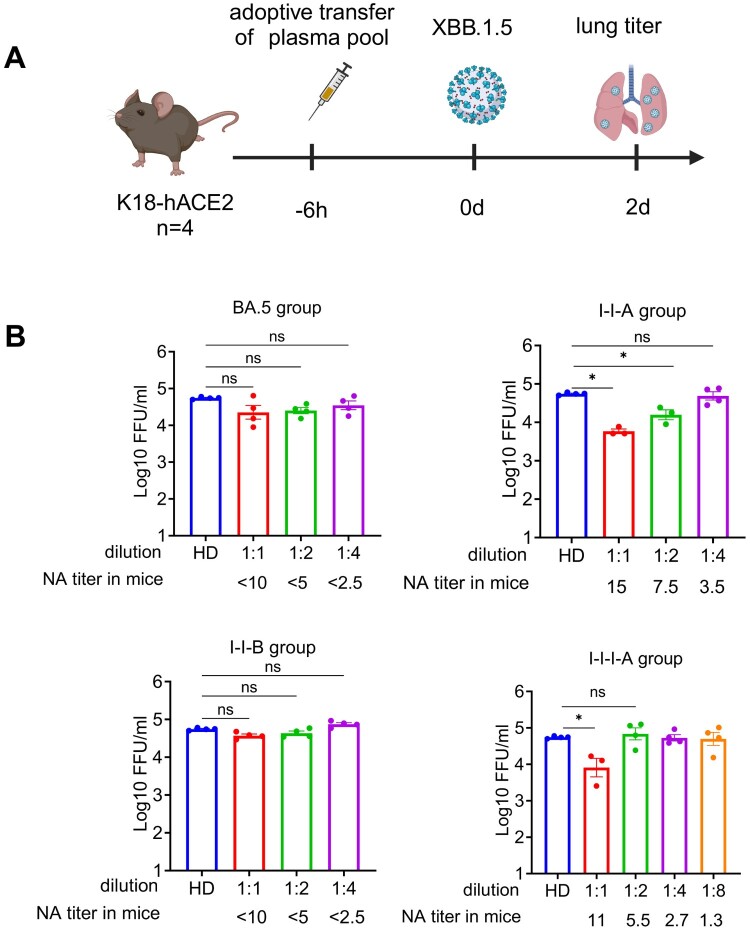
Confirmation of the minimum antibody level required for effective *in vivo* protection using diverse plasma pools. (a) We transferred 200 μL of 2-fold serial dilution of plasma from BA.5, I-I-B, and I-I-I-A group pools to K18-hACE2 mice sensitized for SARS-CoV-2 infection, respectively. Six hours after plasma transfer, challenge the mice with SARS-CoV-2 XBB.1.5. Created in https://BioRender.com. (b) Lung viral titres were measured at 2 dpi. Kruskal–Wallis test with Dunn’s multiple comparisons test was used for evaluating differences among the experimental groups. Error bars represent mean ± SEM, *p*-values are displayed as ns for *p* > 0.05, *p < 0.05, **p < 0.01, ***p < 0.001, and ****p < 0.0001.

To further validate the protective antibody thresholds for SARS-CoV-2 in different cohorts with diverse immune backgrounds, similar plasma adoptive transfer experiments followed by mouse challenge were conducted (Figure 6a). As shown in Figure 6(b), plasma transferred into K18-hACE2 mice intravenously effectively reduce viral titre of lung and protect mice from SARS-CoV-2 infection, effective protection was achieved with a similar minimum antibody titre of pVNT_50_ > 7.5, reinforcing the finding that higher neutralizing antibody titres correlate with greater protection. Collectively, a pVNT_50_ > 7 was defined as the minimum antibody level required to confer effective protection against SARS-CoV-2 infection.

## Discussion

Understanding the protective threshold values for vaccines is crucial for assessing vaccine efficacy and individual immune competence. These thresholds help determine whether an individual has an adequate immune response to protect against infection. Our study aimed to establish the minimum effective protective neutralizing antibody titre threshold against SARS-CoV-2 by evaluating plasma from different vaccine-immunized cohorts and convalescent individuals infected with various SARS-CoV-2 variants. Neutralizing antibody titre is a key measure of the immune response to SARS-CoV-2, as they inhibit the virus from infecting cells, providing a direct measure of immunity. This objective was achieved through a combination of in vitro neutralization assays and *in vivo* studies, which included plasma adoptive transfer followed by viral challenge in mice.

The in vitro neutralization assays evaluated the neutralizing activity of plasma samples against several representative SARS-CoV-2 variants, including wild-type (WT), BA.5, XBB.1, XBB.1.5, XBB.1.16, XBB.2.3, and EG.5. We found that the immune evasion capabilities of the Omicron variants are continuously increasing, as demonstrated by the recent subvariants JN.1, KP.2, and KP.3 [23–25]. These assays provided critical insights into the efficacy of protective antibody response against SARS-CoV-2 variants among populations with diverse immune backgrounds and this study underscores the critical need for updating vaccine strains to match circulating variants. Due to the inherent complexity of plasma, it contains both neutralizing and non-neutralizing antibodies. Both types of antibodies, in addition to direct neutralization, can utilize their Fc domain to mediate effector functions. In this study, we focused on the role of neutralizing antibodies during SARS-CoV-2 infection in mice. While we did not assess the specific contribution of ADCC and ADCP to the overall protective effect in mice, recent studies have shown that ADCC was elicited by 10 days post-infection in humans, peaked between 11 and 20 days, and remained detectable for up to 400 days post-infection. This suggests that the ADCC effect can be long-term and maintained after infection [26]. Additionally, non-neutralizing antibodies targeting the S2 apex, capable of mediating ADCC or ADCP, have been shown to improve survival and reduce lung viral titres in mouse models [27]. However, the protective efficacy of different antibodies mediated by Fc effects varies. In future studies, the contribution of ADCC and ADCP to the protective effects in mice could be evaluated using specific non-neutralizing antibodies rather than plasma. This approach would allow for a more precise understanding of the role of Fc-mediated effector functions in conferring protection.

To establish a more precise protective threshold, we conducted *in vivo* studies. Protective thresholds for SARS-CoV-2 in animal models provide critical insights into the immune response needed to prevent infection and disease. Non-human primates (NHPs) and mice are commonly utilized to evaluate vaccine the efficacy and determine the correlates of protection. Previous studies have demonstrated that human IgG can bind to mouse Fcγ receptors (Fcγ-Rs) and mediate ADCC and ADCP effects with mouse cells [28]. COVID-19 convalescent plasma therapy has been shown to improve the survival of SARS-CoV-2-challenged K18-hACE2 mice [26]. However, potential physiological differences between humans and mice may influence the binding efficiency of Fc receptors. Future studies employing transgenic mouse models expressing human Fcγ receptors will be essential for accurately confirming these effects. In this study, plasma was administered intravenously to mice, which were subsequently challenged with a standardized dose of SARS-CoV-2. We monitored viral titres in the lungs and assessed lung pathology to evaluate the effectiveness of the transferred plasma in preventing infection and reducing viral load. Our findings indicated that plasma with a neutralizing titre (pVNT_50_) of approximately 7 *in vivo* was able to confer protection by significantly reducing lung viral titres.

T cell responses play a complementary role in influencing the protective threshold of neutralizing antibodies. In this study, we employed plasma adoptive transfer from different cohorts into mice to determine the lower threshold of neutralizing antibody titres required for effective protection against SARS-CoV-2 infections. While less commonly quantified as a single threshold value, a robust T cell response significantly contributes to long-term immunity and cross-variant protection. T cell immunity tends to persist longer than antibody responses, offering protection even as antibody titres wane [22]. Previous study has shown that SARS-CoV-2-specific CD8^+^ and CD4^+^ T cells responses were identified in COVID-19 convalescent patients and may play an important role in recovery [29]. Additionally, our recent research demonstrated that SARS-CoV-2 induced cross-reactive T cell responses between SARS-CoV and SARS-CoV-2 in mice [30]. While neutralizing antibodies are essential for preventing viral entry and initial infection, T cells contribute to controlling and clearing infections, especially when antibody levels fall below the protective threshold. The interplay between T cells and neutralizing antibodies has important implications for the durability and breadth of immunity [31].

Previous reports suggest that several factors can influence the protective threshold of vaccines, including assay variability, population differences, and vaccine type [32,33]. Variability in protective thresholds may arise from differences in neutralizing antibody titre assay protocols across different laboratories. Additionally, factors such as age, underlying health conditions, prior exposure to the virus, and genetic factors can impact an individual's immune response and the associated protective thresholds. The specific formulation of the vaccine (e.g. mRNA, inactivated) also affects the immune response and protective threshold. The dose–response relationship between antibody titres and protective effects is shaped by multiple interacting factors. Future studies should aim to integrate these variables to better define protective thresholds and optimize vaccine strategies against SARS-CoV-2 and its variants.

Collectively, our research establishes a neutralizing antibody titre threshold of approximately pVNT_50_ = 7 as the minimum required for effective protection against SARS-CoV-2 *in vivo*, involving a combination of animal models, neutralization assays, and Fc-mediated effector function evaluations. This finding has significant implications for vaccine design and public health strategies, particularly in the context of emerging variants. Future studies should continue to refine these thresholds and explore the longevity and breadth of protection in real-world settings.

## Supplementary Material

20250112 supplementary figures.pdf
