# Supplementary material for: In vivo determination of protective antibody thresholds for SARS-CoV-2 variants using mouse models
Source: Emerg Microbes Infect. 2025 Jan 24;14(1):2459140. doi: 10.1080/22221751.2025.2459140 (PMC11809195; doi:10.1080/22221751.2025.2459140)
Supplement: 20250112 supplementary figures.pdf [file TEMI_A_2459140_SM6326.pdf]

Fig. S1

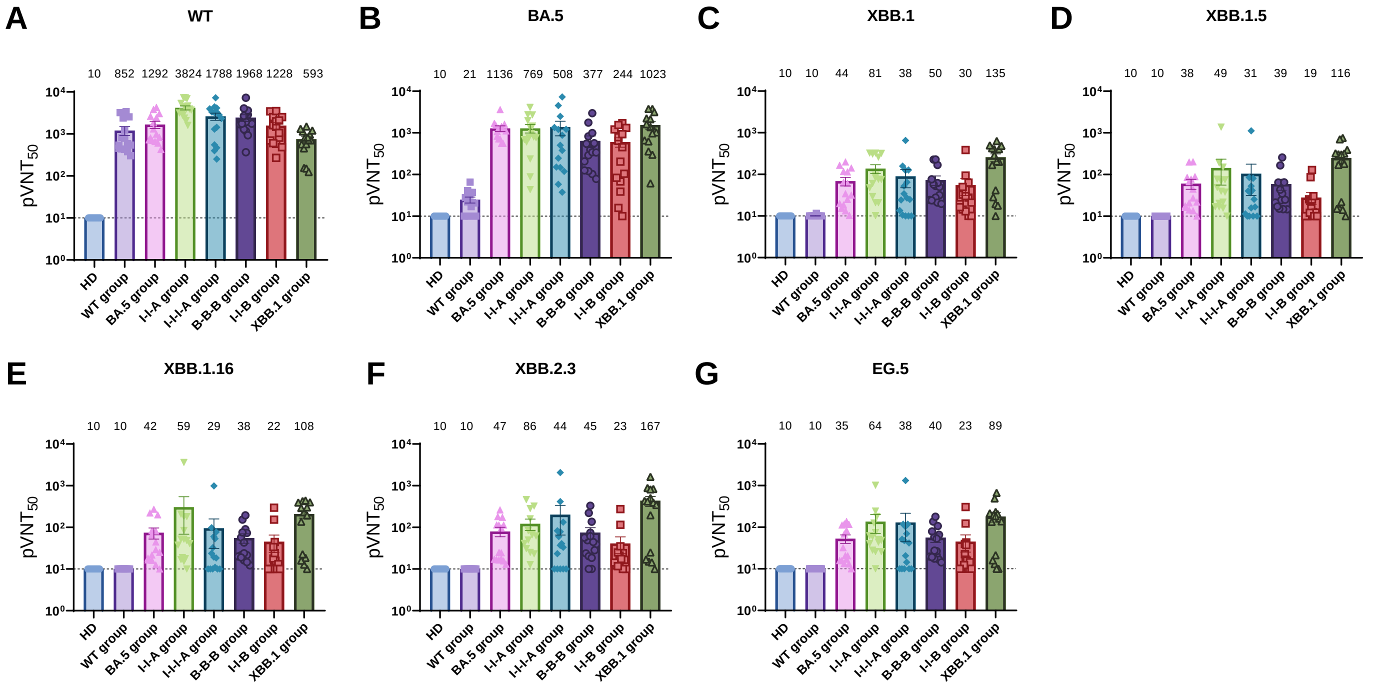

Fig. S1. The comparison of plasma immune escape properties against diverse Omicron subvariants from vaccinees, convalescents and breakthrough infection, Comparison of neutralizing antibody titer against five emerging Omicron subvariants, including WT, BA.5, XBB.1, XBB.1.5, XBB.1.16, XBB.2.3 and EG.5 among all groups. Eight groups participate in this study including HD, WT, BA.5, XBB.1, I-I-A, I-I-I-A, B-B-B and I-I-B group. Geometric means neutralizing titers (GMTs) are displayed at the top of the plots. Limit of detection, $pVNT_{50}$=10.

Fig. S2

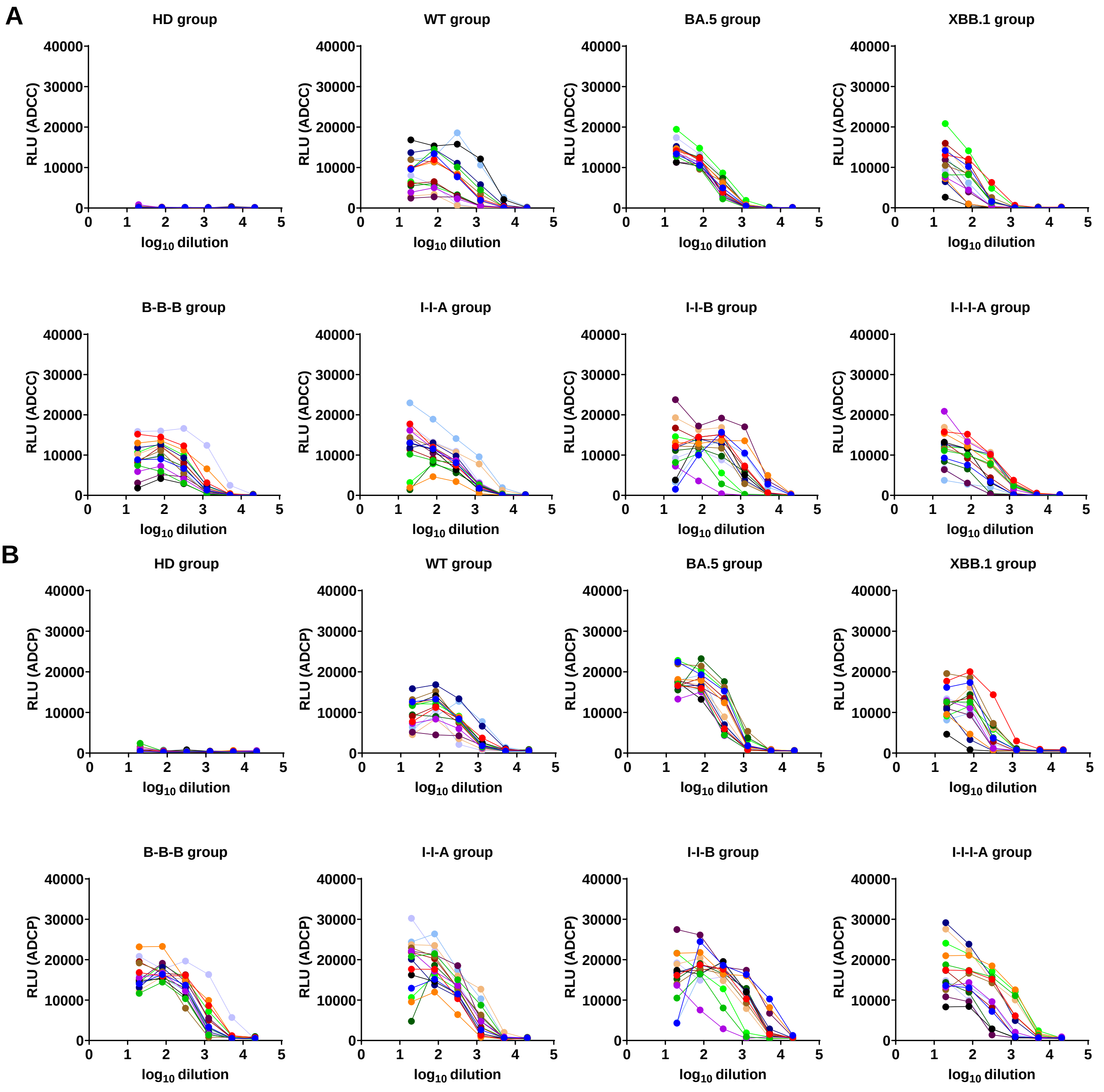

Fig. S2. Antibody-dependent cellular cytotoxicity and phagocytosis induced in different immune groups. (A) Antibody-dependent cellular cytotoxicity (ADCC) induced in HD group, WT group, BA.5 group, and XBB.1 group, B-B-B group, I-I-B group, I-I-A group and I-I-I-A group. **(B)** Antibody-dependent cellular phagocytosis (ADCP) induced in HD group, WT group, BA.5 group, and XBB.1 group, B-B-B group, I-I-B group, I-I-A group and I-I-I-A group.
